# SOCIOECONOMIC, BIOLOGICAL, AND COMMUNITY ASSOCIATIONS WITH OLD-AGE MORTALITY IN CHINA

**DOI:** 10.1093/geroni/igz038.2245

**Published:** 2019-11-08

**Authors:** Yuan Zhang, John A Strauss, Peifeng Hu, yaohui Zhao, Eileen M Crimmins

**Affiliations:** 1 University of North Carolina at Chapel Hill, Chapel Hill, North Carolina, United States; 2 University of Southern California, Los Angeles, California, United States; 3 UCLA, Los Angeles, California, United States; 4 Peking University, Beijing, Beijing, China (People’s Republic)

## Abstract

Determinants of mortality may differ depending on context. This study uses a nationally representative sample of persons aged 60 and over in China to determine if socioeconomic factors, early life conditions, community characteristics, biological and physical functioning, and disease burden predict four-year all-cause mortality. We employed a series of Weibull hazard models based on exact survival time to predict mortality. We find that current education and place of residence, physical functioning, uncontrolled hypertension, diabetes, cancer, a high level of systemic inflammation, and poor kidney functioning are strong predictors of mortality among older Chinese. We did not find linkages between early-life experiences or community infrastructure to mortality at older ages. Results from this study highlight the value of incorporating biological and performance measurements and the importance of social and historical context in studying old-age mortality.

